# Determinants of health-related quality of life among residents with and without COPD in a historically industrialised area

**DOI:** 10.1007/s00420-014-1008-8

**Published:** 2014-12-14

**Authors:** David Fishwick, Leon Lewis, Anthony Darby, Charlotte Young, Ruth Wiggans, Judith Waterhouse, Jeremy Wight, Paul D. Blanc

**Affiliations:** 1 Health and Safety Laboratory, Centre for Workplace Health, Harpur Hill, Buxton, Derbyshire SK173JN UK; 2 University of California, San Francisco, CA USA; 3 University of Gothenburg, Gothenburg, Sweden

**Keywords:** COPD, Health-related quality of life, Work, Job exposure matrix

## Abstract

**Purpose:**

Chronic obstructive pulmonary disease (COPD) is associated with substantial morbidity, including impaired health-related quality of life (HRQoL). Despite the prominent role of occupational factors in the aetiology of COPD, the relationship between these exposures and HRQoL has not been well elucidated.

**Methods:**

A subpopulation from an epidemiological study, designed to assess the workplace contribution to COPD, was administered the EQ5D HRQoL tool. Demographics, an index of economic deprivation, health endpoints including the presence of COPD and lung function were also recorded. Workplace exposures were categorised using both self-reported exposures and also by the use of an established job exposure matrix (JEM).

**Results:**

A total of 623 individuals participated (mean age 67.1 years). One hundred and forty-eight (24 %) reported having received a physician diagnosis of COPD, 355 (57 %) were male, and 386 (62 %) were ever smokers. As anticipated, the presence of COPD was associated with a poorer HRQoL. Additionally, however, HRQoL was significantly lower in the presence of both self-reported vapours, gases, dusts and fumes exposure and JEM-based exposure irrespective of the presence of COPD. Regression analysis, adjusting for a variety of covariates including the presence of COPD, confirmed a persisting higher likelihood of occupational exposure categorised by JEM being associated with poorer HRQoL scores (*β* estimate: −0.069; *p* < 0.05).

**Conclusions:**

Our findings suggest that work may have an important link to HRQoL and that this effect can persist even among those who have retired. In those with COPD, HRQoL is worse than among those without this condition, but the work-associated decrement appears to be similar across both groups.

## Introduction

Chronic obstructive pulmonary disease (COPD) is a common respiratory condition associated with substantial morbidity, including impaired health-related quality of life (HRQoL), (Almagro and Castro 2013; Mahler 2000; Jones and Agusti 2006). While the most important causative agent for COPD remains tobacco smoke, other aetiological factors are increasingly being recognised and quantified. The harmful effects of inhaled occupational exposures have been consistently identified to be associated, on average, with 15 % of the burden of this disease (American Thoracic Society Statement 2003; Darby et al. 2012; Blanc and Torén 2007). Despite the prominent role of occupational factors in the aetiology of COPD, the relationship between such exposures and HRQoL as a COPD outcome has not been well elucidated.

We wished to study the relationship between occupational exposures and HRQoL in those with and without self-reported COPD, using the EuroQol 5 dimension 3 level tool (The EuroQol Group 2013) (EQ-5D-3L™; subsequently referred to as EQ5D). This generic HRQoL instrument has been used in the study of COPD and performs well in comparison with other generic and disease-specific measures. The EQ5D has a key advantage in being a brief battery of questions and related visual analogue scale (VAS) that can yield health utility scores to inform economic assessments of the impact of disease (Brooks et al. 2003; Menn et al. 2010).

We hypothesised that occupational exposures to vapours, gases, dusts and fume (VGDF), stratified either by self-report or assessed through a job exposure matrix (JEM), would be linked to poorer HRQoL in COPD, because such exposures would be likely to increase the likelihood of work disability in the presence of such disease. We further wished to test whether the relationship between these exposures and poorer HRQoL was limited to those with COPD alone or also might be manifested among those without such lung disease.

To address these questions, data were analysed from a population-based study of respiratory disease in a historically industrialised part of the UK. Because this study originally had been designed to assess the contribution of workplace exposures to COPD prevalence, it collected information on both HRQoL and exposure to VGDF among persons with and without COPD (the former over a range of disease severity), thus allowing us to test the hypothesis of interest.

## Materials and methods

This study was carried out as part of a larger epidemiological study of a randomly selected population of Sheffield (UK) residents over the age of 55 years. The details of this larger study are published elsewhere in detail (Darby et al. 2012). In brief, the study used a rolling system to recruit a selected population of Sheffield North residents. The sampling frame was drawn randomly from patient records held in a primary, or community, care database. A second sampling frame, designed to supplement COPD case prevalence in the study population, was drawn from a tertiary care respiratory physiology department.

All participants were asked to self-complete a structured questionnaire including items eliciting demographic information, self-reported respiratory symptoms, self-reported physician-made diagnoses of various respiratory conditions (including asthma, COPD, emphysema and chronic bronchitis), smoking history (yielding a pack-years cumulative exposure), longest held occupation and self-reported previous or current workplace exposures to VGDF. The inclusion of the latter questionnaire item was based on its consistent ability to identify the workplace exposures of interest (Blanc and Torén 2007). In addition to self-reported VGDF, work-related exposure likelihood was assessed using a COPD-specific JEM based on longest held job. This categorised each subject into jobs that were either of low or moderate-to-high likelihood of exposure to inhalants associated with COPD risk (Blanc et al. 2005).

Those agreeing to participate in this phase of the study underwent lung function assessment and completed an additional questionnaire during a home visit. The forced expiratory volume in one second (FEV_1_) and forced vital capacity (FVC) were measured and for each participant, according to the most recent American Thoracic Society and European Respiratory Society guidelines (American Thoracic Society/European Respiratory Society 2005). This was done using a standard, verified, rotameter-based portable spirometer (Microlab, MicroMedical, Rochester, UK). As the lung function testing was carried out at home, bronchodilators were not given prior to lung function testing.

During these home visits, each participant also self-completed the EQ5D. This was comprised of five items assessing different health-related domains (mobility, self-care, usual activities, pain/discomfort and anxiety/depression), each having three levels of response (no problems, some/moderate problems and extreme problems/inability to perform tasks). The scores from these domains can be used to generate a single summary utility index (in the UK validated against the VAS) by applying scores from the EQ5D preference weights measured in general population samples. In addition, a separate EQ5D vertical VAS was administered to record the current health state of the participant; ranging from the “worst imaginable health state” to the “best imaginable health state”.

To assess socio-economic deprivation (SED) for each study participant, the percentage of households within the same postcode (generally containing 10–12 residences) receiving Income Support (%IS), a form of UK-based financial benefit, was used. Previous UK-based work supports such an approach (Danesh et al. 1999).

Data were analysed using SPSS (Version 14). Univariate analysis estimated the associations among the variables of potential study interest and EQ5D scores for each domain, the overall score and the VAS. Based on these findings, multivariate linear regression models were constructed taking into account both COPD and work-related VGDF exposure (self-reported and JEM assigned) as independent predictors of EQ5D, including other key covariates as well. Additional analyses were also performed stratified by the presence or absence of COPD.

In the entire study group, the independent predictor variables of a priori study interest were as follows: age, sex, %IS, pack-years of smoking (calculated from average daily number of cigarettes smoked and duration of smoking), ever previously exposed to VGDF at work (using both self-reported and JEM assignment), FEV_1_ as a percentage of the predicted value (Quanjer et al. 1983) (%FEV_1_) and a self-reported physician diagnosis of COPD. Although in the analysis stratified by presence or absence of COPD that variable was excluded as an independent covariate, the percentage predicted FEV_1_ was retained in the models.

Estimates of the effect of each independent variable was estimated using the unstandardised β coefficient only and its associated 95 % confidence limit (95 % CI). These were not converted to standardised coefficients, in order to allow direct understanding of the effect of the predictor variable in its own units. The overall fit of the model was assessed by the *R* ^2^ metric. The associations of %IS, age, pack-years of smoking, and FEV_1 _% predicted were quantified per 10 units (i.e. per 10 years of age, etc.). The final regression output was assessed for normality by developing a frequency plot of the standardised regression residuals, ensuring that this approximated to a normal distribution.

This study received approval from both the NHS Research Ethics Committee of the UK (IRAS; Integrated Research Application System) and the Sheffield Teaching Hospitals NHS Foundation Trust Research and Development Office.

## Results

Of 2001 initial questionnaire respondents, 623 individuals completed the EQ5D and underwent lung function assessment in home visits (Table 1). Of these, 148 (24 %) reported having received a physician’s diagnosis of COPD, 105 from the general population sample and 43 from the lung physiology laboratory-based recruitment.

**Table 1:** Characteristics among 623 study participants stratified by COPD status

Subject demographics, work exposures and quality-of-life responses	All (*n* = 623)	COPD (*n* = 148)	Non-COPD (*n* = 475)	p*
Male [*n* (%)]	355 (57)	92 (62.2)	263 (55.4)	0.16^a^
Age in years [mean (SD)]	67.1 (8.0)	69.4 (8.2)	66.4 (7.7)	<0.05^b^
% Numbers of individuals receiving income support in postcode area [mean (SD)]	18.6 (16.4)	22.8 (14.9) (*n* = 103)	17.6 (16.6) (*n* = 427)	<0.05^a^
Ever smoker [*n* (%)]	386 (62.1)	126 (85.1) (*n* = 147)	260 (54.7) (*n* = 474)	<0.05^a^
Ever VGDF exposed [*n* (%)]	368 (59.3)	117 (79.1) (*n* = 143)	251 (52.8) (*n* = 464)	<0.05^a^
JEM exposure 2 or 3 [*n* (%)]	326 (52.3 %)	95 (64 %)	231 (48.6 %)	<0.05^a^
Mean FEV_1_ in litres [Mean (SD)]	2.20 (0.77)	1.65 (0.7)	2.37 (0.7)	<0.05^b^
Equation 5D VAS [Mean (SD)]	70.8 (19.1)	57.0 (16.5) (*n* = 146)	75 (17.8) (*n* = 472)	<0.05^b^
Utility index [Mean (SD)]	0.68 (0.31)	0.52 (0.32)	0.73 (0.30)	<0.05^b^

For participants with missing responses, study (*n*) presented

*SD* standard deviation, *VGDF* vapours, gases, dust or fume exposure, *JEM* job exposure matrix, *FEV*_*1*_ forced expiratory volume in 1 s, *EQ5D VAS* EuroQol five dimension visual analogue scale quality-of-life measure

Utility index; summary score derived from a combination of the 5 EQ5D domains, used as a single measure of HRQoL with a maximum value of 1.0 (1.0 representing the best QOL)

This is done by applying scores from the EQ5D preference weights measured in general population samples

* *p* value compares Chronic Obstructive Pulmonary Disease (COPD) and non-COPD groups

^a^Chi-squared tests (or Fisher’s exact equivalent)

^b^Independent samples *t* test

The majority of the participants (355; 57 %) was male, and 386 (62 %) were ever smokers. The mean age of the participants was 67.1 years, and the mean level of %IS for the group was 14.3 %. Of the entire study population, a minority of the 149 (24 %) were currently working at the time of study. The majority (368; 59 %) reported ever having been regularly exposed to VGDF in their longest held job, while 326 (52.3 %) were classified based on the JEM as having been in a longest held job with likely exposure.

Table 1 shows further data on demographics, lung function, disability and HRQoL, stratified by the presence or absence of a reported physician’s diagnosis of COPD. The 148 individuals with COPD were older, had higher levels of socioeconomic deprivation, were more likely to be past or current smokers, were less likely to be currently employed and, consistent with their reported physician’s diagnosis, on average had 720 mls lower FEV_1_ (all *p* < 0.01). Adverse occupational factors, whether by self-report of VGDF or by JEM, were more prevalent in the COPD stratum. The EQ5D VAS score was significantly lower in the group with COPD. There was also a parallel decrement in the numeric EQ5D composite utility index comparing those with and without self-reported COPD.

The presence of self-reported COPD also was associated with a poorer HRQoL as assessed by each of the five EQ5D domains (data not in Table). For example, of those with self-reported COPD, 80 % reported any problem with mobility (combining the two higher categories and excluding no problem), 34 % for self-care, 74 % for usual activities, 78 % for pain and discomfort and 46 % for anxiety/depression. The respective figures for those without self-reported COPD were as follows: mobility 44 %, self-care 11 %, usual activities 36 %, pain and discomfort 58 %, and anxiety/depression 26 %.

Table 2 details the EQ5D domain and total scores stratified in two ways: by self-reported VGDF exposure and exposures assessed by JEM. As shown, HRQoL was significantly lower in the presence of both self-reported VGDF exposure and JEM-based exposure. Particularly high levels of reported pain and discomfort were seen within both the JEM highly exposed and VGDF-exposed strata. Overall mean utility index and VAS scores indicated exposure-associated poorer HRQoL.

**Table 2:** EQ5D domain scores stratified by JEM and VGDF exposures

Equation 5D dimension		JEM		*p* value	VGDF		*p* value
		Not exposed	Exposed		VGDF	No VGDF	
Mobility	Level 1	161	134		147	141	
	Level 2–3	136	192	<0.05*	221	98	<0.05*
Self-care	Level 1	254	261		291	212	
	Level 2–3	41	64	0.06*	74	27	<0.05*
Usual activities	Level 1	179	161		172	158	
	Level 2–3	118	165	<0.05*	196	81	<0.05*
Pain discomfort	Level 1	128	105	<0.05*	114	114	
	Level 2–3	169	221		254	125	<0.05*
Anxiety/depression	Level 1	208	218	0.42*	237	179	
	Level 2–3	87	105		126	60	<0.05*
Mean VAS score		72.8	68.9	<0.05**	67.7	75.0	<0.05**
Mean utility index		0.72	0.65	<0.05**	0.64	0.75	<0.05**

Higher EQ5D dimension levels 2–3 indicate poorer quality of life than level 1

*VGDF* vapours, gases, dust or fume exposure, *JEM* job exposure matrix

* *p* values derived by Chi-squared testing

** *p* values from independent samples *t* test

Table 3 shows the results of the multiple linear regression analysis carried out for 564 of the study population (137 with COPD, 427 without COPD), representing all those with complete data for all key variables. Older age, female sex, greater levels of socioeconomic deprivation, lower percentage predicted FEV_1_ and a reported diagnosis of COPD were significantly associated with poorer HRQoL. Taking all of these covariates into account in the multivariate model, a higher likelihood of occupational exposure by JEM remained associated with poorer HRQoL (*β* estimate: −0.069; *p* < 0.05).

**Table 3:** Multivariate analysis of predictors of health-related quality of life (EQ5D) among 564 study participants

Explanatory variables	Univariate analysis unstandardised regression coefficient			Multivariate analysis unstandardised regression coefficient		
	*β*	95 % CI		*β*	95 % CI	
Percentage income support (per 10 % increment)	−0.06	−0.07	−0.04	−0.03	−0.05	−0.02
Age (years, per 10 years)	−0.08	−0.11	−0.05	−0.05	−0.08	−0.02
Female	−0.049	−0.099	0.001	−0.121	−0.174	−0.068
Pack-years (per 10 pack-years)	−0.03	−0.04	−0.02	−0.01	−0.02	0.0005
JEM exposure	−0.076	−0.126	−0.027	−0.069	−0.123	−0.016
FEV_1 _% predicted (per 10 %)	0.04	0.03	0.05	0.02	0.01	0.03
Self-reported COPD	−0.207	−0.263	−0.151	−0.090	−0.155	−0.026

Dependent variable is the EQ5D utility index

Adjusted models are mutually adjusted for all variables in the model

All associations *p* < 0.05 except female in unadjusted model (p = 0.056) and pack-years in the adjusted model (*p* = 0.06)

Utility index; summary score derived from a combination of the 5 EQ5D domains, used as a single measure of HRQoL with a maximum value of 1 (1 representing the best HRQoL). This is done by applying scores from the EQ5D preference weights measured in general population samples

*JEM* job exposures matrix-based exposure (see Methods)

A further analysis including the variable “currently working” found a positive association with HRQoL of being currently employed, while estimates for the remaining covariates remained similar to the previous model (data not shown). Additional analysis of the association between occupational exposure defined by JEM but using EQ5D measured with the VAS instrument as the dependent variable, including the same covariates, yielded a negative direction of effect consistent with that seen with using point scale EQ5D measure, but the relationship was not statistically significant (*β* = −2.9; *p* = 0.074) (data not shown in Table).

Table 4 shows the results of a subsequent regression analysis stratified by the presence or absence of self-reported COPD. Within the self-reported COPD group, the negative effect of JEM exposure manifested wider confidence intervals including zero and hence nonsignificant, but the point estimate of the negative effect (*β* = −0.070) was similar to the entire group and to the non-COPD stratum, in which the association remained statistically significant. The estimates for the relationship between pack-years of smoking and HRQoL were also similar across the strata. Of note, the magnitude the SES effect appeared to be greater among those in the COPD stratum.

**Table 4:** Multiple regression analysis of independent predictors of health-related quality of life using the EQ5D utility index stratified by COPD status

Explanatory variables	Unstandardised *β* coefficient	*p* value	95 % confidence interval for *β*	
			Lower limit	Upper limit
*COPD* *(n* *=* *137)*				
Percentage income support per postcode (per 10 % change)	−0.02	0.35	−0.05	0.02
Age (per 10 years)	0.002	0.61	−0.05	0.08
Female	−0.167	<0.05	−0.283	−0.050
Pack-years (per 10 pack year units)	−0.02	0.07	−0.03	0.000
JEM Exposure	−0.070	0.26	−0.192	0.051
*No COPD* *(n* *=* *427)*				
Percentage income support per postcode (per 10 % change)	−0.04	<0.05	−0.06	−0.03
Age (per 10 years)	−0.07	<0.05	−0.11	−0.04
Female	−0.096	<0.05	−0.155	−0.037
Pack-years (per 10 pack year units)	−0.02	<0.05	−0.03	−0.0003
JEM exposure	−0.061	<0.05	−0.121	−0.020

For the EQ5D utility index, summary scores are derived from a combination of the 5 EQ5D domains, used as a single measure of HRQoL with a maximum value of 1 (1 representing the best QOL) (see Methods)

*JEM* job exposure matrix

In an alternate model (identical to Table 3 but using self-reported VGDF in place of JEM), in the entire group exposure by this measure did have a negative association with HRQoL (*β* = −0.109, *p* < 0.05). Similarly, when stratified by COPD status (identical to Table 4 but using VGDF rather than JEM), exposure by this measure again had a negative association with HRQoL (*β* = −0.199, *p* < 0.05) for those with COPD and also for those without COPD (*β* = −0.081, *p* < 0.05).

All standardised regression residual plots, for the regression data shown, approximated to a normal distribution.

## Discussion

Although this population-based study was designed to estimate the contribution of occupational exposures to the presence of self-reported COPD as its primary endpoint, it provided the opportunity to analyse occupational factors in relation to HRQoL as a secondary outcome measure. We found that attributes of subjects’ longest held job, specifically the likelihood of inhalant exposure based on a JEM assignment and self-report of exposure to VGDF, were associated with poorer HRQoL, This was true even taking into account demographics, a self-reported physician’s diagnosis of COPD, and lung function. Moreover, occupational exposure defined by JEM was associated with poorer HRQoL even among those without COPD, with similar estimates of effect to that of the COPD stratum. Although the latter point estimate was similar, the 95 % CI included zero. Because the measure of HRQoL we used was not respiratory specific, this suggests that poorer working conditions may impact HRQoL in ways not limited to adverse effects on the lung. Notably, the association with self-reported VGDF, unlike the JEM-based exposure measure, was stronger in the COPD stratum (*b* = −0.199, *p* < 0.05), consistent with a reporting bias (those with disease and poorer HRQoL may have been more likely to report VGDF exposure). In terms of the analysis confined to the COPD stratum, only the effect of gender persisted significantly following adjusted analyses. The potential predictor variables included in the analysis we believe are valid. These variables had predicted HRQoL in the study group as a whole, and it was for this reason that they were retained in the analysis of the COPD only stratum.

The possible relationship between adverse outcomes in COPD being related to previous workplace-based exposures has been raised to our knowledge very uncommonly in the literature. A single previous study identified a relationship between having both reported VGDF exposures and respiratory-related work disability as predicting the greatest risk at 12 months follow-up for restricted activity days attributed to a breathing or lung problem, although this study did not report HRQoL (Blanc et al. 2004).

The limitations of this analysis should temper the interpretation of our findings. This was a cross-sectional analysis precluding causal inference. Further, these data are taken from a study whose primary intent was to assess the association between COPD and occupational exposure. The association between self-reported VGDF and poorer HRQoL, as noted above, is likely to be impacted by reporting bias. This would not explain, however, the association we observed between HRQoL and JEM-based exposure, which was not affected by recall of exposure. Moreover, JEM-based exposure manifested a similar relation to HRQoL among those with and without COPD, although the effect within the COPD stratum was not statistically significant. Because the EQ5D is a generic rather than respiratory specific HRQoL measure, we cannot argue that its relationship to exposures on the longest held job reflects adverse respiratory effects *per se* and, furthermore, the association was present even taking COPD diagnosis and lung function into account in multivariate modelling. Were jobs with poorer working conditions simply a surrogate for lower SES, we would have expected adjustment for neighbourhood deprivation to have substantially weakened the association we observed, which was not the case. Of interest, there was indeed a lower SES-associated negative association with HRQoL, although this appeared to be driven by an association within the COPD stratum.

Selection effects of those with ill health could have influenced our findings and may limit their generalisability. Arguing against this, the mean EQ5D VAS score of 80 (Interquartile range; IQR 29.5) within the stratum of our study population without self-reported COPD is similar to reported population norms of 83.7, 78.2 and 72.9 (males), and 80.3, 76.6 and 74.2 (females) for age groups 55–65, 64–75, and 75+, respectively (Szende et al. 2014). We also observed similarly consistent findings with the question-based EQ5D instrument. Our study size is relatively modest and accounts for the wide confidence intervals observed, especially within the COPD stratum. We also acknowledge that the definition of COPD we applied (self-reported physician diagnosed COPD, including COPD, emphysema or chronic bronchitis) may have led to misclassification error. By including in our multivariate analysis FEV_1_ per cent predicted, we accounted for this in part, as well as capturing disease severity within the COPD group. Although this may have led to a degree of “over-adjustment” insofar as COPD is concerned, it should not explain the association with HRQoL that we did observe for occupational inhalant exposure on the longest held job. Finally, we cannot exclude unaccounted for confounding between job exposures and some other factor or factors driving poorer HRQoL. For example, those exposed by JEM or VGDF may have been in certain job types that increased the risk of mental ill health or selected for such problems.

Despite these potential limitations, our findings suggest that work may have an important link to HRQoL and that this can persist even among those who have retired. In COPD, HRQoL is even worse than among those without this condition, but the work-associated decrement appears to be similar across the COPD and non-COPD strata, at least in terms of the generic measure we used. It will be important to further elucidate these relations, including with disease-specific HRQoL measures, in longitudinal studies. This will require, however, considerable accumulated follow-up, well past the span of active labour force participation, in order to fully assess the potential impact of work on the key patient-centred outcome of quality of life.
